# Analysis of goal, feedback and rewards on sustained attention via machine learning

**DOI:** 10.3389/fnbeh.2024.1386723

**Published:** 2024-12-19

**Authors:** Nethali Fernando, Matthew Robison, Pedro D. Maia

**Affiliations:** ^1^Department of Mathematics, University of Texas at Arlington, Arlington, TX, United States; ^2^Division of Data Science, College of Science, University of Texas at Arlington, Arlington, TX, United States; ^3^Department of Psychology, University of Notre Dame, Notre Dame, IN, United States; ^4^Department of Psychology, University of Texas at Arlington, Arlington, TX, United States

**Keywords:** sustained attention, pupillometry data, reward, reaction time, machine learning, classification, regression, feature selection

## Abstract

**Introduction:**

Sustaining attention is a notoriously difficult task as shown in a recent experiment where reaction times (RTs) and pupillometry data were recorded from 350 subjects in a 30-min vigilance task. Subjects were also presented with different types of goal, feedback, and reward.

**Methods:**

In this study, we revisit this experimental data and solve three families of machine learning problems: (i) RT-regression problems, to predict subjects' RTs using all available data, (ii) RT-classification problems, to classify responses more broadly as attentive, semi-attentive, and inattentive, and (iii) to predict the subjects' experimental conditions from physiological data.

**Results:**

After establishing that regressing RTs is in general a difficult task, we achieve better results classifying them in broader categories. We also successfully disambiguate subjects who received goals and rewards from those who did not. Finally, we quantify changes in accuracy when coarser features (averaged throughout multiple trials) are used. Interestingly, the machine learning pipeline selects different features depending on their resolution, suggesting that predictive physiological features are also resolution-specific.

**Discussion:**

These findings highlight the potential of machine learning to advance research on sustained attention and behavior, particularly in studies incorporating pupillometry or other physiological measurements, offering new avenues for understanding and analysis.

## 1 Introduction

### 1.1 Background

Sustained attention is an umbrella term used in the field of cognitive psychology generally referring to a subject's readiness to detect unpredictably occurring signals over prolonged periods (Sarter et al., 2001). Studies dating back from over a century have addressed the difficulties of remaining vigilant when performing trivial or repetitive tasks (Bills, 1931a,b; Thorndike, 1912). Since then, several authors have explored the pitfalls of human inattentiveness in a variety of contexts, from driving accidents (National Highway Traffic Safety Administration, 2020) to in-class pedagogical activities (Bligh, 2000; Wang et al., 2021). This remains an active research topic in experimental psychology, with ongoing efforts to identify effective strategies to mitigate decrements in vigilance and task engagement (Massar et al., 2016; Hopstaken et al., 2015a,b; Esterman et al., 2016).

In a recent study, Robison et al. (2021) examined the specific effects of goal-setting, feedback, and incentives to counter attention deficits in a psychomotor vigilance task. Although some of the experimental manipulations were effective in mitigating slower Reaction Times (RTs) at later trials, none eliminated it. Specifically, both providing participants with a specific goal to strive toward and giving them performance feedback mitigated the a worsening of task performance across time. Further, participants in these conditions self-reported feeling more motivated and exhibited greater evoked pupillary responses. Traditionally, pupil diameter has been used as a measure of attentional effort (Beatty, 1982). In recent years, pupillometry has become an increasingly common psychophysiological tool, as we learn more about its neurobiological derivations and the psychological processes it accompanies. For example, it has been demonstrated that the pupil is sensitive to cognitive conflict (van der Wel and van Steenbergen, 2018), momentary lapses of attention (Unsworth and Robison, 2016, 2018), successful/unsuccessful memory retrieval (Papesh et al., 2012; Goldinger and Papesh, 2012), and can even be used to determine whether or not an individual is mind-wandering (Franklin et al., 2013; Mittner et al., 2016). Further, eye-gaze based detection of mind-wandering has been successfully employed while people read (Foulsham et al., 2013), watch films and video lectures (Mills et al., 2016; Hutt et al., 2017) and engage with online learning systems (Hutt et al., 2019). Therefore, we had reason to believe we could leverage state-of-the-art machine learning tools to predict an individual's psychological state, momentary level of attentiveness, and behavior via pupillometry and gaze positions during a simple sustained attention task.

### 1.2 Objectives

The objective of this work is to revisit the extensive pupillometry and reaction time (RT) data collected in Robison et al. (2021) and create new predictive models of attentiveness using a modern machine learning (ML) framework. In ML, a typical model (estimator) starts with a set of preprocessed features (explanatory variables) and is trained to predict a target variable from these features. If the target variable is continuous, the task is considered a regression problem. If the target variable is categorical, the task is a classification problem. The richness of the original study allows us to propose multiple ML problems of interest by varying the corresponding target variable and the composition of the feature set. We will solve:

1. RT-regression problems: for these models, the target variable is the continuous, raw RT value. The differences in problems arise from the initial feature list, which can vary in size from 12 to 360 features.
2. RT-classification problems: for these models, each RT value is categorized into attentive, semi-attentive, or inattentive. Broadly classifying responses in this manner is more feasible than predicting exact RT values. In these models, the number of initial features can vary from 7 to 12.
3. EC-classification problems: for this third family of problems, we propose a radically different approach. In the previous problems, the experimental conditions (EC) were used as explanatory variables. However, in these problems, the ECs become the target variables. For instance, we study whether it is possible to predict, based on the RTs, whether a subject received an incentive or not.

RT predictions similar to the first family of problems were attempted in Robison et al. (2021). However, here we explore various initial feature lists and regressors available in scikit-learn. The last two families of ML problems have not been explored yet. The outline of the paper is as follows: First, we review the experimental setup of the psychomotor vigilance task from Robison et al. (2021), describing how eye measurements were collected throughout the trials and detailing the various experimental manipulations that subjects underwent, which could impact their motivation and, consequently, their RTs. Next, we explain our pipeline for pre-processing the features that will be used in the subsequent models and carefully define the three families of machine learning problems investigated in our study. Third, we describe the feature matrices and pipelines used in each case. In the results section, we summarize the balanced accuracy scores and *R*-squared values for all classification and regression problems, along with a ranking of features according to their importance. Finally, we discuss and interpret our results in light of modern psychological theories.

## 2 Methods

### 2.1 Psychomotor vigilance task

**Figure 1:**
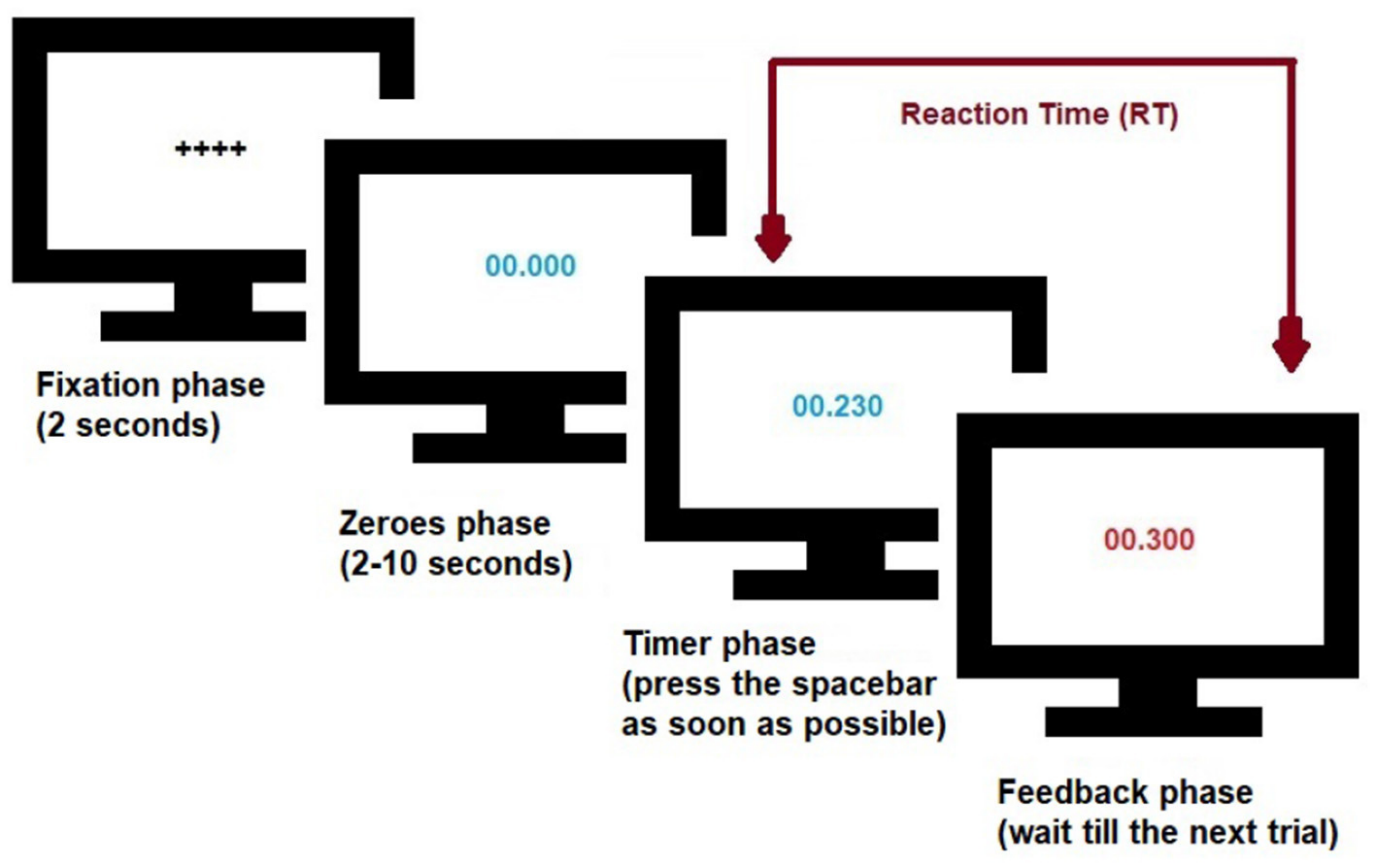
Schematics of the Psychomotor vigilance task. Screen undergoes different phases, changing from fixation, zeroes, timer, to feedback. The zeroes phase consists of a random interval of 2–10 s. Subjects were asked to press the space bar as soon as the stopwatch starts and have their Reaction Time (RT) recorded for that trial. See Robison et al. (2021) and text for details.

Figure 1 illustrates the psychomotor vigilance task described in Robison et al. (2021). Briefly, subjects (*N* = 353) in a dark room stare at a blank screen that is first replaced by a fixation screen and then by a screen that displays zeroes for a random interval between 2–10 s. After this delay, the zeroes begin counting forward like a stopwatch. Subjects were instructed to press the space bar as soon as the stopwatch starts (timer phase). Once pressed, the counting stops, the screen freezes for 1 s (in some cases displaying a feedback screen), the RT is recorded, the screen resets, and a new trial begins. The subject's eye movements (diameter of left/right pupils and their gaze positions) are recorded throughout the process (140 trials).

As listed in Table 1 (top left), the experimental conditions varied among subjects in terms of the type of goal (easy, hard, or none), feedback (yes/no), and reward conditions (time incentive, cash incentive, or none). Subjects in group *G*_0_ were asked to respond to the cue as quickly as possible. For subjects in group *G*_1_, the average reaction time (RT) was provided every 28 trials. Subjects in group *G*_2_ were asked to keep their RT below 800 ms (easy goal). Subjects in groups *G*_3_ and *G*_4_ were asked to keep their RT below 300 ms (hard goal), with *G*_4_ subjects receiving the same feedback as those in *G*_1_. Subjects in group *G*_5_ were told they could leave early if they met the goal, while group *G*_6_ subjects were promised $10 upon reaching the goal. In reality, all groups completed the same number of trials, regardless of performance. Table 1 (right) lists all measurements collected during the experiment and the derived features used in our study.

**Table 1:** Subject groups, reaction-time labels, and (derived) features.

	**Group**	**Goal**	**Feedback**	**Reward**	**Subjects**	**Measurement**	**Features**	**Description**	
**Exp. conditions**	G0	No	No	No	68	Subject IDs	Subject IDs	348 categorical variables to encode different IDs	**Data**
	G1	No	Yes	No	34	Trial number	Trial number	Integers ranging from 1–140	
	G2	Easy	No	No	34	Pupil diameter		Time series recording of right eye's pupil diameter (in mm) throughout trial	
	G3	Hard	No	No	70		Mean_DIAM	Mean of the right pupil diameter.	
	G4	Hard	Yes	No	71		Var_DIAM	Var. of the right pupil diameter.	
	G5	Hard	Yes	Time	35	Left-eye Gaze		Time series recording of left eye's gaze in *x*−*y* directions	
	G6	Hard	Yes	Cash	37		Mean_LDEV	Mean of left eye's deviation	
							Var_LDEV	Variance of left eye's deviation.	
						Right-eye Gaze		Time series recording of right eye's gaze in *x*−*y* directions	
	**Problem**	**Clustering Method**	**Attentive (Att.)**	**Semi-Attentive (semi-Att.)**	**Inattentive (Inatt.)**		mean_LDEV	Mean of right eye's deviation.	
**RTs labels**	$C_{RT}^{pop}\left[1\right]$	GMM	83.3% RT < 441	15.7% 441 < RT < 769	0.01% RT>769		var_LDEV	Variance of right eye's deviation.	
	$C_{RT}^{pop}\left[2\right]$	KNN	84.1% RT < 445	15.6% 445 < RT < 1,207	0.003% RT < 1,207	Exp. Conditions		Goal (3), Feedback (2), Reward (3)	
	$C_{RT}^{pop}\left[3\right]$	Naive	33.4% RT < 330	33.3% 330 < RT < 386	33.2% RT < 386		Dummy_vars	5 categorical variables to encode different ECs	

**(Left top)** Experimental Conditions (EC) varied from subject to subject, giving rise to seven different patient EC groups (*G*0, …*G*6). **(Left bottom)** Reaction Time (RT) responses were labeled as attentive, semi-attentive or inattentive according to three methods (GMM, KNN and Naive method). The RT range for the labels are also presented (see text for details). (Right) Measurements and derived features used in all regression and classification problems are also described.

### 2.2 Description of the problems

In our study, we solve three families of machine learning problems: (i) four RT-regression problems at the population level, (ii) three RT-classification problems at both individual and population levels, and (iii) six Experimental Condition (EC) classification problems at group levels. See Table 2 for details.

**Table 2:** Description of the machine learning problems.

**Problem**		**Label**	**Comparisons**	**Features**	**Initial feature list**
**RT regression**	$R_{RT}^{pop}\left[1\right]$	–	–	349	*348 subject IDs^a^ + trial no*.
	$R_{RT}^{pop}\left[2\right]$	–	–	355	*348 subject IDs + trial no. + 6 eye features*.
	$R_{RT}^{pop}\left[3\right]$	-	–	360	*as above + 5 EC variables.^b^*
	$R_{RT}^{pop}\left[4\right]$	–	–	12	*trial no. + 6 eye features + 5 EC variables*.
**RT three-way classification**	$C_{RT}^{pop}\left[1\right]$	via GMM	[Att.] × [Semi-att.] × [Inatt.]	12	*trial no. + 6 eye features + 5 EC variables*.
	$C_{RT}^{pop}\left[2\right]$	via KNN	[Att.] × [Semi-att.] × [Inatt.]	12	*Trial no. + 6 eye features + 5 EC variables*.
	$C_{RT}^{pop}\left[3\right]$	Naive	[Att.] × [Semi-att.] × [Inatt.]	12	*Trial no. + 6 eye features + 5 EC variables*.
	$C_{RT}^{ind}\left[1\right]$	via GMM	[Att.] × [Semi-att.] × [Inatt.]	7	*Trial no. + 6 eye features*.
	$C_{RT}^{ind}\left[2\right]$	via KNN	[Att.] × [Semi-att.] × [Inatt.]	7	*Trial no. + 6 eye features*.
	$C_{RT}^{ind}\left[3\right]$	Naive	[Att.] × [Semi-att.] × [Inatt.]	7	*Trial no. + 6 eye features*.
**EC classification**	$C_{EC}^{group}\left[1\right]$	Binary Goal	[G0] x [G2 + G3]	980	[RT + 6 eye features] x 140 trials
	$C_{EC}^{group}\left[2\right]$	Binary feedback	[G0] × [G1]	980	[RT + 6 eye features] × 140 trials
	$C_{EC}^{group}\left[3\right]$	Binary reward	[G4] × [G5 + G6]	980	[RT + 6 eye features] × 140 trials
	$C_{EC}^{group}\left[4\right]$	Tertiary goal	[G0] × [G2] × [G3]	980	[RT + 6 eye features] × 140 trials
	$C_{EC}^{group}\left[5\right]$	Tertiary reward	[G4] × [G5] × [G6]	980	[RT + 6 eye features] × 140 trials
	$C_{EC}^{group}\left[6\right]$	All seven subgroups	[G0] × [G1] × ... × [G6]	980	[RT + 6 eye features] × 140 trials

For RT regression and RT three-way classification problems we use data collected in fixation phase only. For EC classification problems we use the data from the full trial. In our notation, *R* and *C* stand for Regression and Classification problems respectively. The subscripts *RT* and *EC* indicate if the goal of the problem is predict the Reaction Time or the experimental conditions used to classify the subjects into their groups. Finally, the superscripts *pop, ind*, and *group* stand for population, individual or group levels respectively.

^a^Subject ID's are dummy variables.

^b^EC variables are dummy variables.

#### 2.2.1 RT-regression problems $R_{RT}^{pop}\left[1,\ldots ,4\right]$

For task (i), we regress reaction times at the population level using the experimental conditions and pupillometry data collected during the fixation phase. As shown in Table 2, the initial feature list varies between problems 1–4, containing 349, 355, 360, and 12 features respectively. This is a challenging ML task because we do not use eye measurements collected during the random interval (2–10 s) between the fixation phase and the start of the stopwatch that records the RT for the trial.

#### 2.2.2 RT-classification problems $C_{RT}^{pop}\left[1,2,3\right]$ and $C_{RT}^{ind}\left[1,2,3\right]$

For (ii), we classify RT responses into three classes: attentive, semi- attentive, and inattentive using three labeling methods listed in Table 1: Gaussian Mixture Model (GMM), K-Nearest Neighbors (KNN), and the naive method (see Supplementary material for details). This can be viewed as a coarser reformulation of the regression problems, where instead of predicting the exact RT values we try to predict broader RT categories. These classification problem are solved both at the population (pop) level and at the individual (ind) level. Here again, we do not utilize any eye measurements collected during the zeroes phase.

#### 2.2.3 EC-classification problems $C_{EC}^{group}\left[1,\ldots ,6\right]$

For (iii), we classify subjects according to labels given by experimental conditions. We use pupillometry data collected throughout the entire experiment as well as RTs as features to predict the label. The labels are based on the subject's group and the classification problem that we solve. For some binary classification problems, for example, we merge two groups into one label. See Table 2 for details.

### 2.3 Machine-learning pipelines

**Figure 2:**
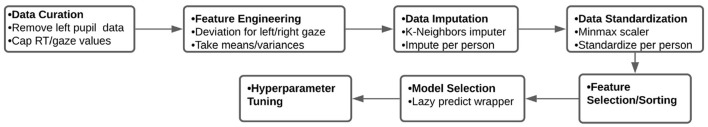
Overview of the machine learning pipeline. We tailor this general outline for the RT-regression problems, for the RT three-way classification problems, and for the EC-classification problems. See text for details.

Figure 2 depicts the seven key steps in the general pipeline used in the regression and classification problems: data curation, feature engineering, data imputation, data standardization, feature selection, model selection, and hyper parameter tuning. See the paragraphs below for some details.

#### 2.3.1 Data curation and feature engineering

We curate the data by (i) removing subjects 1,041, 1,191, 1,192, and 1,205 who lacked eye-related measurements. (ii) by capping RT values at 3,000 ms to avoid outlying RTs from having an outsized influence on the results. To avoid redundancies in the feature list, we disregard the left-pupil measurements as they are highly correlated with the right-pupil ones. See the pairwise correlation matrix between the original features in Supplementary Figure 8. We also combine the eye gazes in the *x*−*y* directions (capped at 10 mm) to create an overall deviation feature denoting how much they deviate from the center of the screen located at (0.5, 0.5):


(1) $$\begin{array}{l} \text{Deviation}=\sqrt{{\left(\text{Gaze x}-0.5\right)}^{2}+{\left(\text{Gaze y}-0.5\right)}^{2}} \end{array}$$

The eye-tracker used in this study (Tobii T300) reports the position of visual gaze using a coordinate system where (0.5, 0.5) represents the center of the screen (half the screen width, half the screen height). Since numerical values are standardized later in the pipeline, this choice of coordinate system does not affect the results. For each subject, we calculate the means and variances of all eye-derived features throughout trials.

#### 2.3.2 Data imputation and standardization

The filling of missing eye data is done using sklearn's k-neighbors imputer, and values are subsequently standardized (individually) to stay within a 0–1 range via min-max scaler.

#### 2.3.3 Inclusion of categorical data in feature list

We use dummy encoding to account for categorical data (subject IDs and experimental conditions) in the RT-regression and classification problems. This is not necessary for the EC-classification problems as they only consider numerical features.

#### 2.3.4 Wrapper for multiple classifiers and regressors

There are numerous models to solve classification and regression problems. Instead of manually implementing each model, we use a wrapper called *lazy predict* (Pandala, 2021) that automatically tests 42 regressors or 32 classifiers available in scikit-learn (Pedregosa et al., 2011) to your data. This allows us to automatically get *R*-squared values or balanced accuracy scores for all listed models. We adjust the original wrapper to also return the Akaike Information Criterion (AIC) values to help us select the most informative models. Since the wrapper results may vary depending on the splitting of the train/test subsets, we perform five-fold cross-validation and consider the mean of the results.

#### 2.3.5 Hyperparameter tuning

Once we identify the best model using the lazy wrapper, we further optimize all parameters used by the chosen regressor or classifier to improve the results. We report our final *R*-squared and balanced accuracy scores for five-fold (stratified) cross validations.

#### 2.3.6 Pipeline adjustments for RT-problems

We use step-wise backward regression in our feature selection step for problems $R_{RT}^{pop}\left[1,\ldots ,3\right]$, $C_{RT}^{pop}\left[1,2,3\right]$, and $C_{RT}^{ind}\left[1,2,3\right]$. These problems have initially fewer features compared to the EC-classification problems, and this choice provides us with a reasonable number of features to work with. $R_{RT}^{pop}\left[1,2,4\right]$ problems use *p*-value = 0.05, but $R_{RT}^{pop}\left[3\right]$ uses *p*-value = 0.9 as the algorithm fails to converge at lower values. We use a more stringent *p*-value of 0.01 for $C_{RT}^{pop}\left[1,\ldots ,3\right]$. For the individual level classification problems, we start with a *p*-value = 0.05, but we relax this value (up to 50%) for three subjects who did not have any features selected.

#### 2.3.7 Pipeline adjustments for EC-problems

Problems $C_{EC}^{group}\left[1,\ldots ,6\right]$ have initially a large number of features (*n* = 980), which we first reduce and sort using a combination of LASSO lars and Ridge regression. See Supplementary material for details. We run the wrapper five times (with different train/test splittings of the data) for every subset of features. We select the classifier that provides the highest (mean) balanced accuracy and lowest (mean) AIC to avoid overfitting,

## 3 Results

### 3.1 Summary of results

Table 3 summarizes the results for the three families of machine learning problems that we solve: (i) four RT-regression problems at the population level $R_{RT}^{pop}\left[1,\ldots ,4\right]$, (ii) six RT-classification problems $C_{RT}^{pop}\left[1,2,3\right]$ and $C_{RT}^{ind}\left[1,2,3\right]$, and (iii) six experimental Condition (EC) classification problems at group levels $C_{EC}^{group}\left[1,\ldots ,6\right]$. We list the number of features before and after feature selection, the best classifier or regressor for each problem, and the corresponding balanced accuracy or *R*-squared score. We favor balanced accuracy scores over (simple) accuracy for all classification problems to account for differences in group sizes for the different labels. Note that the scores for all classification problems surpass random guessing by a significant margin.

**Table 3:** Summary of results for regression and classification problems.

**Problem**		**Labels**	**Initial Features**	**Selected No. of Features**	**Class./Reg. Method**	***R* ^2^**	**Balanced Accuracy**	**Random Guess**	**AIC**	**CrossVals**.
**RT regression**	$R_{RT}^{pop}\left[1\right]$	–	349	232	MLPRegressor	**0.24**	**-**	**-**	273083	6
	$R_{RT}^{pop}\left[2\right]$	–	355	237	MLPRegressor	**0.25**	**–**	**–**	272,620	6
	$R_{RT}^{pop}\left[3\right]$	–	360	343	MLPRegressor	**0.25**	**–**	**–**	272,888	6
	$R_{RT}^{pop}\left[4\right]$	–	12	9	MLPRegressor	**0.04**	**–**	**–**	279,952	6
**RT three-way classification**	$C_{RT}^{pop}\left[1\right]$	via GMM	12	7	Nearest centroid	**–**	**0.42**	**0.33**	–121,941	20
	$C_{RT}^{pop}\left[2\right]$	via KNN	12	7	Nearest centroid	**–**	**0.44**	**0.33**	–121,998	20
	$C_{RT}^{pop}\left[3\right]$	Naive	12	10	Linear Dis. analysis	**–**	**0.42**	**0.33**	–116,086	20
	$C_{RT}^{ind}\left[1\right]$	via GMM	7	3	Nearest centroid	**–**	**0.7**	**0.33**	–	5
	$C_{RT}^{ind}\left[2\right]$	via KNN	7	3	Adaboost classifier	**–**	**0.58**	**0.33**	–	5
	$C_{RT}^{ind}\left[3\right]$	Naive	7	2	Adaboost classifier	**–**	**0.49**	**0.33**	–	5
**EC classification**	$C_{EC}^{group}\left[1\right]$	[G0] × [G2 + G3]	980	8	SGD classifier	**–**	**0.69**	**0.5**	–665	400
	$C_{EC}^{group}\left[2\right]$	[G0] × [G1]	980	3	Linear dis. anaysis	**–**	**0.64**	**0.5**	–406	400
	$C_{EC}^{group}\left[3\right]$	[G4] × [G5 + G6]	980	5	Calibrated class. cv	**–**	**0.7**	**0.5**	–549	400
	$C_{EC}^{group}\left[4\right]$	[G0] × [G2] × [G3]	980	8	Ridge classifier	**-**	**0.51**	**0.33**	–616	400
	$C_{EC}^{group}\left[5\right]$	[G4] × [G5] × [G6]	980	8	Linear SVC	**–**	**0.48**	**0.33**	–499	400
	$C_{EC}^{group}\left[6\right]$	[G0] × [G1] × ... × [G6]	980	13	Linear SVC	**–**	**0.24**	**0.14**	–1,069	400

All classification problems beat the random guess. The feature selection method for RT regression and RT three-way classification problems is stepwise-backward. LASSO lars and Ridge regression are used for EC classification problems. For problems, $C_{RT}^{ind}\left[1,\ldots ,3\right]$ selected number of features and the balanced accuracy values are averaged across individuals. The classifier method for these problems is the classifiers that provided the highest median balanced accuracy for the majority of the individuals.

### 3.2 Subject ID as key regressor for predicting RTs at population level

**Figure 3:**
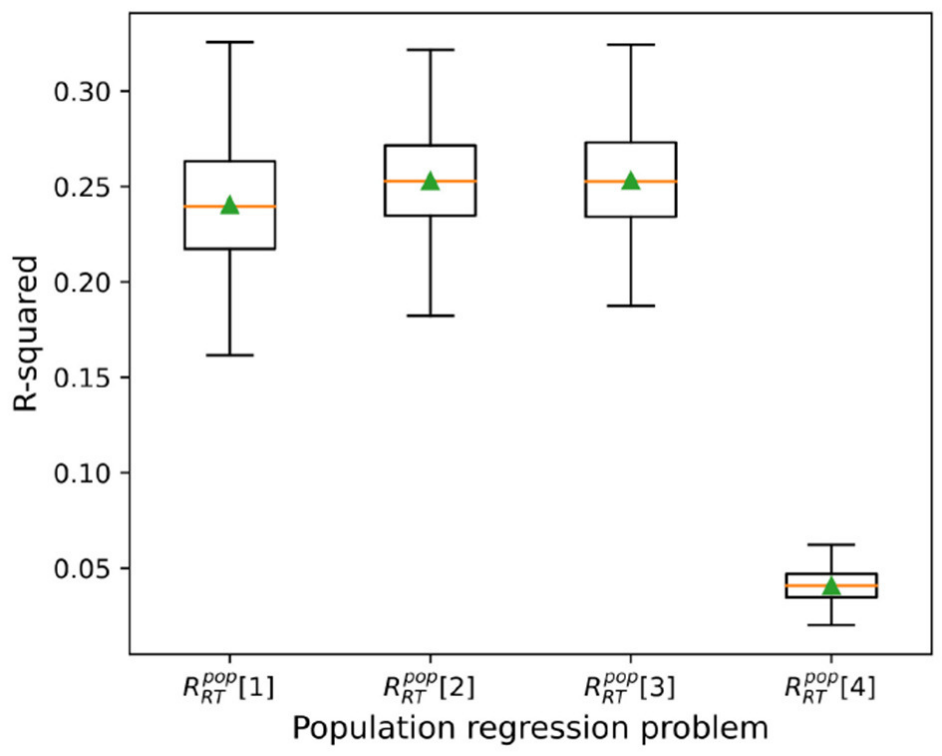
Regression results. Box plots depicting mean, median, and standard deviation of *R*^2^ values in cross validations for $R_{RT}^{pop}\left[1,\ldots ,4\right]$ regression problems.

The box-plots in Figure 3 depicts *R*-squared values over 400 cross-validations for problems $R_{RT}^{pop}\left[1,\ldots ,4\right]$ using our top model, the Multi-layer Perceptron regressor (MLP regressor). Problems $R_{RT}^{pop}\left[1,2,3\right]$ include 348 subject IDs in the initial feature list, and their mean *R*-squared are 0.25 ± 0.03, 0.25 ± 0.03, 0.24 ± 0.03 respectively. Contrasting, the mean *R*-squared for problem $R_{RT}^{pop}\left[4\right]$ (that does not include subject IDs) is 0.04 ± 0.01, demonstrating the importance of IDs as RT predictors. Interestingly, the addition of categorical variables encoding experimental conditions (problem $R_{RT}^{pop}\left[2\right]$ vs. $R_{RT}^{pop}\left[3\right]$) did not lead to any improvement in the predictions.

### 3.3 Results for RT-classification problems

Table 4 lists the selected features for problems $C_{RT}^{pop}\left[1,2,3\right]$. Note that the trial number and the categorical variable encoding the presence of a goal were selected by all problems. All problems select at least one of the pupillometry features and an experimental condition, with the Nearest centroid classifier being the best method for problems $C_{RT}^{pop}\left[1,2\right]$. Many classifiers provide similar balanced accuracy scores for $C_{RT}^{pop}\left[3\right]$ (adaboost, label propagation, linear discriminant, LGBM, random forest, SVC), so we chose linear discriminant as it is simpler to hyper-tune. We run five cross-validations with the wrapper (testing all methods) and 20 cross-validations for the best one.

**Table 4:** Selected features for classification problems.

	**Trial**	**Mean**	**Var**.	**Mean**	**Var**.	**Mean**	**Var**.	**Feed-**	**Goal**	**Goal**	**Reward**
	**no**.	**Diam**.	**Diam**.	**LDEV**	**LDEV**	**RDEV**	**RDEV**	**back**	**1**	**2**	**1**
GMM	✓					✓	✓	✓	✓	✓	✓
KNN	✓					✓	✓	✓	✓	✓	✓
Naive	✓	✓	✓	✓	✓	✓		✓	✓	✓	✓

List of selected features for problems $C_{RT}^{pop}\left[1,\ldots ,4\right]$ with *p*-value ≤ 0.01 in stepwise regression for the three labeling methods. None of the methods selected the dummy variable Reward 2.

**Figure 4:**
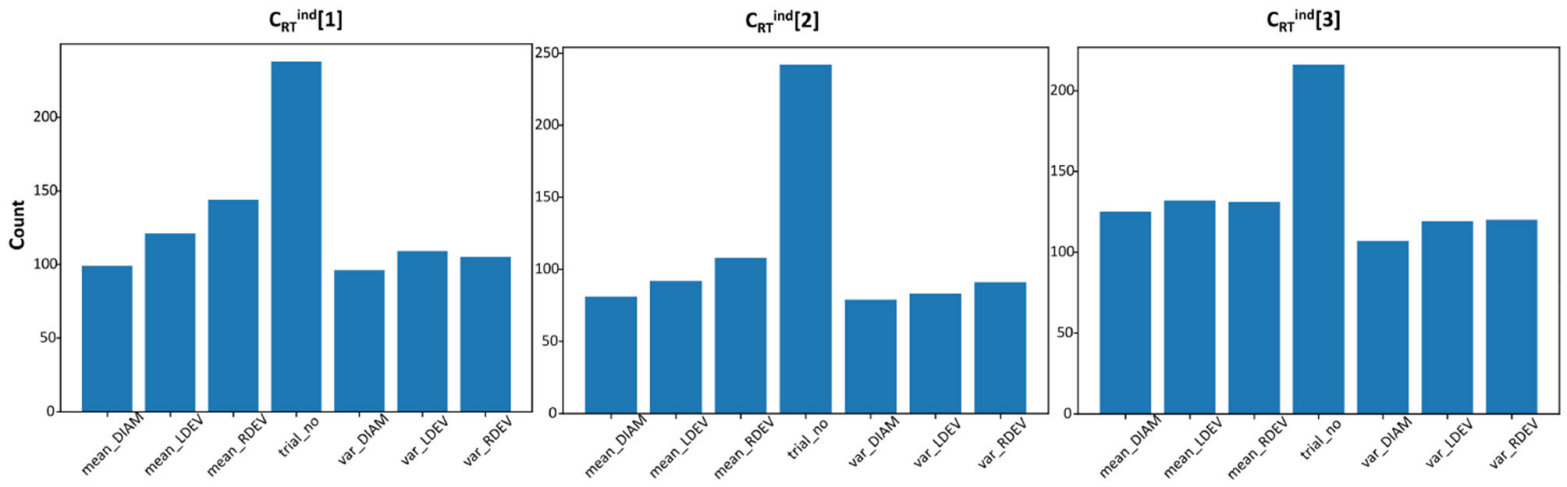
Frequency of features for individual, RT-classification problems $C_{RT}^{ind}\left[1,2,3\right]$ under each clustering method. Majority of the individuals selected less than three out of the seven features.

We solve the RT-classification problems also at the individual level, i.e., trying to classify RT responses as attentive, semi-attentive, and inattentive for each subject in turn. For these problems ($C_{RT}^{ind}\left[1,2,3\right]$), the experimental conditions are not included in the feature list. Table 3 shows the mean of the balanced accuracy scores (using the best method) over all subjects. We run five cross-validations with the wrapper (testing all methods) and rank the methods according to the median of the balanced accuracy scores. Figure 4 shows histograms for the frequency of selected features for problem $C_{RT}^{ind}\left[1,2,3\right]$. Similar to the results obtained at the population level, the trial number was also the most frequently selected feature for most subjects.

### 3.4 Identifying experimental conditions from physiological data

The last family of machine learning problems examined in this study is significantly different from the other two; while $R_{RT}^{pop}\left[1,\ldots ,4\right]$ and $C_{RT}^{pop}\left[1,2,3\right]$ try to predict or categorize RT responses, problems $C_{EC}^{group}\left[1,\ldots ,6\right]$ try to disambiguate subjects exposed to different experimental conditions. As shown in Table 1, subjects were binned into groups *G*_0_, …, *G*_6_ depending on the presented type of goal (easy, hard, or none), feedback (yes/no), and reward (time incentive, cash incentive, or none). For these problems, experimental conditions are no longer features used to predict RTs. In fact, RTs are now themselves features used in the six n-way classification tasks (see label type in Table 2). Also, the eye-derived features are no longer collected solely at the fixation phase of the experiment, but instead, throughout the entire trial.

With this different setup, we can now investigate if differences in experimental conditions imprint noticeable changes in the collected physiological data. In problem $C_{EC}^{group}\left[1\right]$, for example, we combine subjects who received either easy or hard goals into a single group [*G*_2_+*G*_3_] and compare them with those who received none [*G*_0_]. This leads to a binary classification problem, [*G*_0_] × [*G*_2_+*G*_3_]. In problem $C_{EC}^{group}\left[4\right]$, we keep *G*_2_ and *G*_3_ separate, which lead to a three-way classification problem instead, [*G*_0_] × [*G*_2_] × [*G*_3_]. The setup for the other $C_{EC}^{group}$ problems follows the same rationale, and our best classification results are listed in the bottom part of Table 3.

Problems $C_{EC}^{group}\left[1,2,3\right]$ consist of binary classification tasks for one type of experimental condition (vs. control group). Top balanced accuracy scores for the presence of goal, feedback, and reward are 0.69, 0.70, and 0.64 respectively. Problems $C_{EC}^{group}\left[4,5\right]$ consist of three-way classification tasks for goal and reward with top scores of 0.51 and 0.48. Finally, $C_{EC}^{group}\left[6\right]$ is a seven-way classification task for all groups, for which we achieve a 0.24 score. All scores surpass random guessing by a significant margin, and in all problems except $C_{EC}^{group}\left[4\right]$, the most accurate model was also the most informative (using AIC).

**Figure 5:**
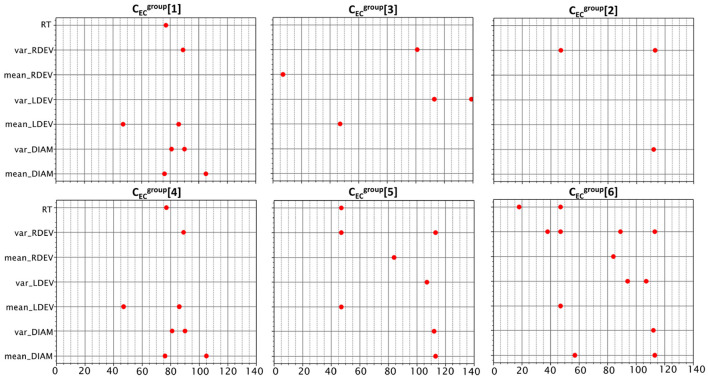
Features selected for the six classification problems. There were common features among the binary and three-way problems of goal and reward experimental conditions. The binary classification problems are on the top row. The three-way problems for goal and reward are placed under their respective binary problems.

Figure 5 depicts the selected features for problems $C_{EC}^{group}\left[1,\ldots ,6\right]$. Since their initial feature list is very large (with 980 features), it is expected that in some cases, the pipeline's original feature-selection step might provide sub-optimal solutions. In an effort to improve our results, we empirically complemented the list of selected features of some problems with features selected by others. Specifically, $C_{EC}^{group}\left[4\right]$ improved when we added features from $C_{EC}^{group}\left[1\right]$, $C_{EC}^{group}\left[5\right]$ improved when we added features from $C_{EC}^{group}\left[2\right]$, and $C_{EC}^{group}\left[6\right]$ improved when we added features from $C_{EC}^{group}\left[2,5\right]$. As expected, the number of features is proportional to the complexity of the task, with significant overlap between the corresponding binary and three-way tasks ($C_{EC}^{group}\left[1,4\right]$ and $C_{EC}^{group}\left[3,5\right]$).

### 3.5 Accuracy vs. feature resolution for EC-classification problems

The initial feature list for problems $C_{EC}^{group}\left[1,\ldots ,6\right]$ is large because we collect six pupillometry features along with the RT per trial (7 × 140 = 980). In what follows, we investigate how well can we classify subjects using coarser measurements. Instead of collecting features at every trial, we average them over 10, 20, and 35 trials, thus obtaining physiological data with decreasing resolution. We use the same feature sorting and selection methods used in the original $C_{EC}^{group}\left[1,\ldots ,6\right]$ problems.

**Figure 6:**
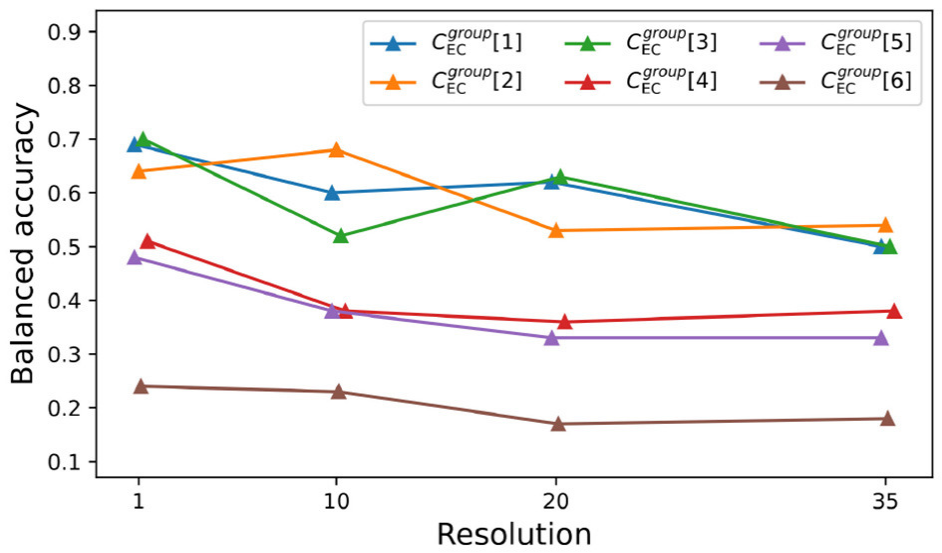
Balanced accuracy for problems at different resolutions. Average balanced accuracy for problems $C_{EC}^{group}\left[1,\ldots ,6\right]$ at different resolution levels (1, 10, 20, and 35). We used repeated Stratified K-Fold with 20 folds and 20 repeats totaling 400 cross-validations. Best fit lines are given by *y*_1_ = −0.006*x* + 0.69, *y*_2_ = −0.005*x* + 0.67, *y*_3_ = −0.005*x* + 0.66, *y*_4_ = −0.004*x*+0.47, *y*_5_ = −0.005*x* + 0.46 and *y*_6_ = –0.002*x* + 0.24 respectively.

Figure 6 displays the mean of the balanced accuracy scores over 400 cross-validations (repeated stratified k fold with 20 splits and 20 repeats) for the best method found by the wrapper. In general, scores tend to slowly decrease as we average features over more trials. Problems $C_{EC}^{group}\left[1,\ldots ,5\right]$ have similar decay rates in balanced accuracy (≈−0.005), while $C_{EC}^{group}\left[6\right]$ is slightly more robust (≈−0.002). We report the number of selected features, the exact scores, the standard deviations, the best classifiers, and the AIC values for all problems (at different resolutions) in the Supplementary material.

**Figure 7:**
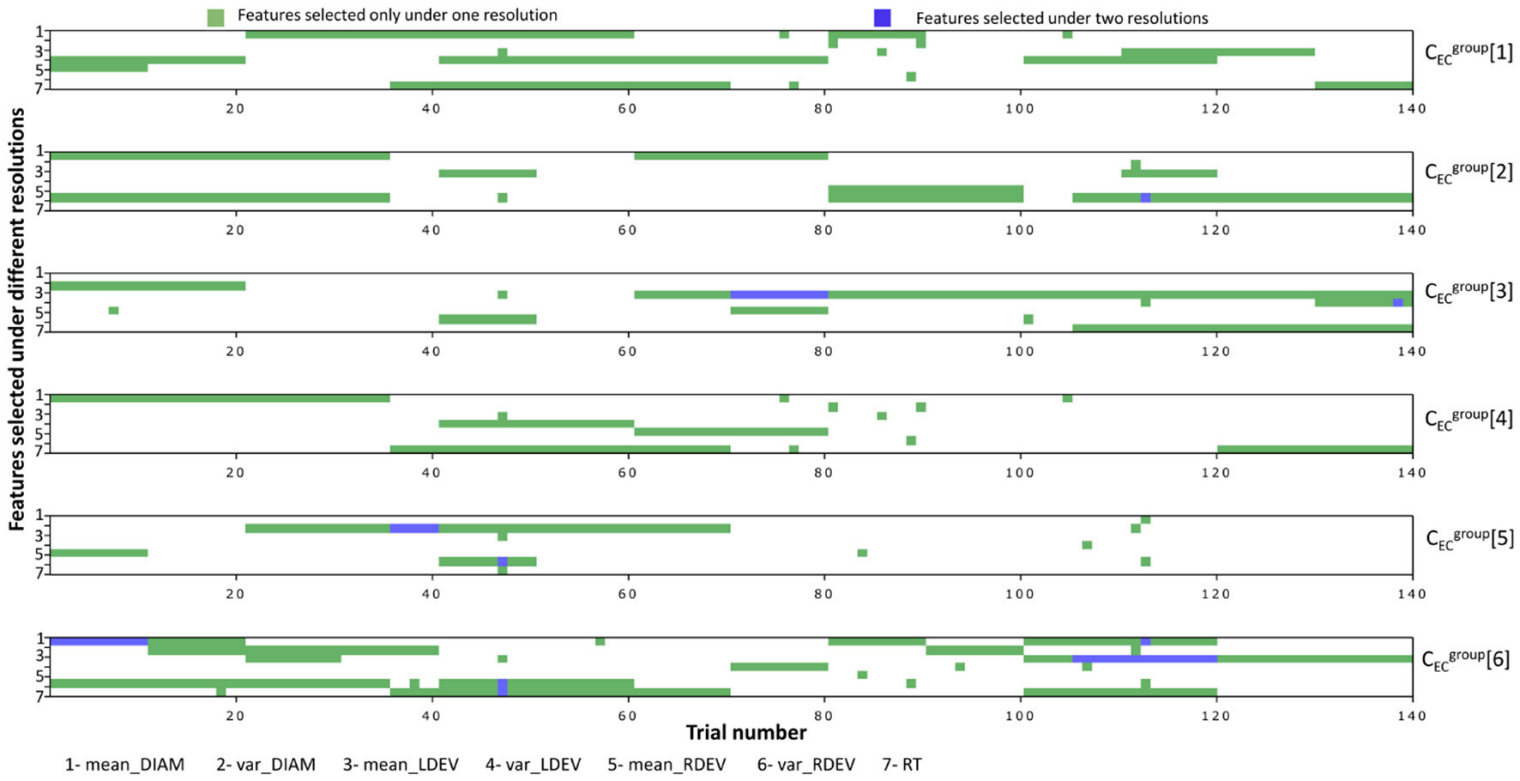
Key features selected for each problem under different resolutions. If a feature was selected for a combined trial (10, 20, and 35), then the whole block was colored. The dark green color indicated overlaps in features in different resolutions.

Figure 7 shows the important features for problems $C_{EC}^{group}\left[1,\ldots ,6\right]$, with green/blue blocks representing features selected once/twice under any resolution level. Moreover, if a selected feature is averaged over k trials, we color all corresponding k trials in the matrix plot. Note that there are no overlaps for more than two resolution levels, indicating that predictive features at the highest resolution level are not optimal at the lower ones.

## 4 Discussion

Sustaining attention is difficult, and it has been made perhaps more difficult by the dynamic environments in which we live and work. As people complete their daily tasks, they experience failures of sustained attention, caused by both internal (e.g., mind-wandering) and external distraction (e.g., from smartphone notifications). Many situations, like driving a car, learning in a classroom, and screening baggage for dangerous items, require sustained attention. Cognitive psychologistsv and cognitive neuroscientists still do not have a firm grasp on precisely why humans find sustaining attention so challenging. Modern machine learning methods may reveal, via data-driven approaches, moment-to-moment psychological and physiological features that are indicative of greater versus letter attentiveness. Our goal here was to determine whether we could categorize attentiveness using both psychological (i.e., goal state, feedback presence, reward) and physiological (i.e., eye-tracking) features of individuals.

Specifically, we revisit the psychomotor vigilance experiment from Robison et al. (2021) through the lens of modern machine learning techniques. Specifically, we solve three families of machine learning problems: (i) four RT-regression problems at the population level $R_{RT}^{pop}\left[1,\ldots ,4\right]$, (ii) three RT-classification problems $C_{RT}^{pop}\left[1,2,3\right]$ at the population level and $C_{RT}^{ind}\left[1,2,3\right]$ at the individual level, and (iii) six Experimental Condition (EC) classification problems at group levels $C_{EC}^{group}\left[1,\ldots ,6\right]$.

In (i), we find that predicting RTs is a notoriously difficult task (*R*-squared ≈0.24, see Table 3). The best models for $R_{RT}^{pop}\left[1,\ldots ,4\right]$ did not select any eye-related features from the fixation phase as predictors, instead choosing a combination of subject IDs, trial number, and experimental conditions. During the experiment, subjects first stared at a blank screen, followed by a fixation screen, and then a screen displaying zeroes for a random interval between 2–10 s. After this delay, the zeroes began counting forward like a stopwatch, and subjects pressed the space bar as soon as the stopwatch started (timer phase). Our analysis utilized eye-derived features from the fixation phase, where subjects could still be attentive and preparing for the task ahead. Any signs of inattention may not have developed yet. In contrast, during the random interval, subjects might experience a drop in attentiveness or their eyes might wander off the screen, which could be more predictive of slower RTs. This interval represents a period of waiting and uncertainty, potentially leading to lapses in attention that could be more indicative of the subject's state and performance. Therefore, future analyses might benefit from incorporating eye-derived features from this interval to improve RT predictions. We also note that including Subject ID in the RT-regression model significantly improved its performance, underscoring the importance of individual differences in experiments on sustained attention using RT measures. This finding aligns with existing literature, which shows that these are nested observations, and RTs naturally cluster within individuals, as some people tend to respond faster than others (Unsworth and Robison, 2018).

In (ii), instead of trying to predict exact RT values, we settle for a less ambitious classification of RT-responses as attentive, semi-attentive, and inattentive. We use four different clustering methods to define these broader categories, which lead to balanced accuracy scores of ≈0.43 at the population level and ≈0.6 at the individual level. The trial number feature was the most frequently-selected feature across all problems (see Figure 4), which is compatible with the natural, growing inattentiveness reported in Robison et al. (2021). The analysis also revealed that pupil and eye-tracking measures were not key predictors of RT in both RT-regression and RT-classification models. This could be due to the restricted time period of eye measures or because RT and pupil measures index different aspects of attention. Existing literature supports the latter possibility, suggesting that post-stimulus dilations and RT have different sensitivities to task demands (Hershman and Henik, 2019; Richer and Beatty, 1987). Therefore, including wider windows of pupil data may not have straightforwardly predicted RTs.

In (iii), we solve a much different type of machine learning problem. Instead of trying to categorize or predict RTs, we try to classify subjects that exposed to different experimental conditions, such as the presence of a goal, feedback, and reward. This led to the six n-way classification problems $C_{EC}^{group}\left[1,\ldots ,6\right]$. Contrary to the previous problems, RTs are now themselves features and eye-measurements are no longer restricted to the fixation phase (they are averaged throughout the entire trial). Our good classification scores (see Table 3) suggest that manipulations in experimental conditions do imprint noticeable changes in the recorded physiological data, although predictors may vary according to the resolution of the features (see Figures 6, 7). The analysis showed that pupil measures are key predictors of task type in the EC-classification problem, with prediction accuracy decreasing when measures are averaged across trials rather than using individual trial-level data. This suggests that while broad pupil/eye data can indicate the overall arousal or attentional state of participants, trial-level data offers a weaker prediction with temporal resolution. We believe this finding may help guide future research methodology in cognitive psychology, reinforcing the importance of maintaining data resolution throughout the experiment.

Overall, our machine-leaning approach indicates that the behavioral and physiological measures we used accurately predicted the experimental conditions, demonstrating that global, qualitative changes in attentional state are detectable. In this design, participants were assigned to various conditions, and it is plausible that similar techniques could predict natural states of extreme stress, anxiety, fatigue, or their opposites like contentment, calm, and alertness. These states would manifest as distinct patterns in eye movements, pupillary dynamics, and behavior. However, the same model might be less effective in predicting specific moments of stress or fatigue, as evidenced by our lower success rate in predicting specific RTs.

### 4.1 Outlook

The application of ML to analyze sustained attention data presents significant theoretical, analytical, and practical benefits. Theoretically, ML allows us to identify complex patterns and relationships within the data, enhancing our understanding of cognitive processes in sustained attention. This analytical approach is particularly valuable in behavioral neuroscience and psychology, providing deeper insights into complex behaviors.

Analytically, ML offers robust and flexible tools to handle high-dimensional data, enabling the development of predictive models with high accuracy and generalizability. Our study demonstrates the potential of these models to classify attentional states and predict RTs. The experimental setup led to multiple ML problems, highlighting the importance of carefully defining problems, as the initial feature list and target variable may change. For the same setup, we proposed three families of ML problems. However, predicting human responses remains challenging, even in controlled setups, illustrating the necessity of sophisticated ML pipelines.

Practically, predicting and understanding attentiveness through ML has far-reaching implications. In education, predictive models can inform personalized learning strategies to maintain engagement. In occupational health, these models can monitor and enhance productivity and safety by identifying periods of low attentiveness and implementing timely interventions. Additionally, adaptive user interfaces and assistive technologies can respond to users' attentional states, improving overall user experience and performance.

The present results are a first step toward leveraging pupil diameter and gaze position data to predict moments of inattentiveness. Recent work has pursued similar goals with more dynamic environments and additional data (e.g., body movement, blinking) (D'Mello et al., 2022; Bosch and D'Mello, 2022). In future work, we aim to design tasks that produce robust indices of inattentiveness using eye data and additional sensors for multidimensional data collection. Ultimately, our goal is to build a reproducible and generalizable model that detects attentional lapses in real time, mitigating failures of sustained attention. Portable and accurate wearable eye-tracking technology could be especially useful in air traffic control, baggage screening, life-guarding, and others.

## Data Availability

The machine learning code supporting this study is available upon reasonable request by contacting netznana@gmail.com. Access to the dataset can be obtained by contacting mrobison@nd.edu.
