# Supplementary material for: Analysis of goal, feedback and rewards on sustained attention via machine learning
Source: Front Behav Neurosci. 2024 Dec 19;18:1386723. doi: 10.3389/fnbeh.2024.1386723 (PMC11701782; doi:10.3389/fnbeh.2024.1386723)
Supplement: Supplementary file 1 [file Data_Sheet_1.pdf]

Supplementary Information (SI)

Correlation between the derived features: Figure S1 illustrates a heat map of the pearson correlations between the original derived characteristics, with lighter colors corresponding to high correlation values.

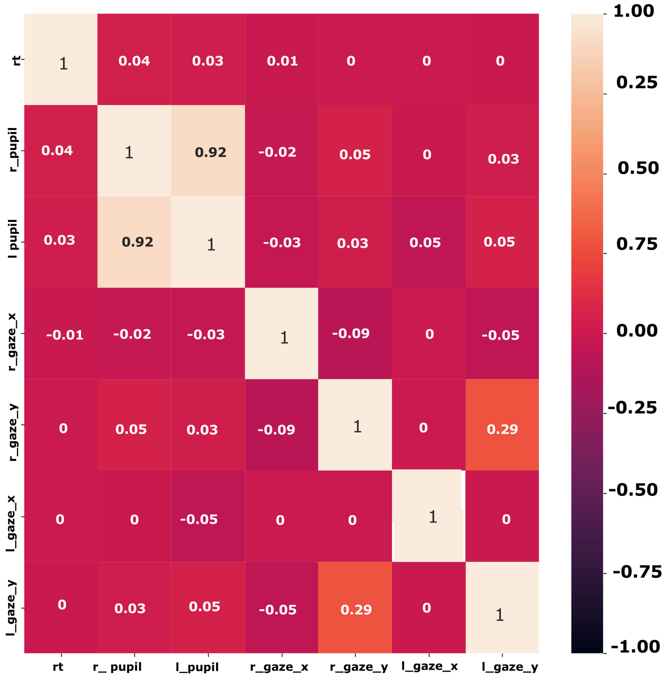

Figure 1: **Matrix with pairwise Pearson correlation between original features**. Here the right and left pupillometry features are abbreviated with r and l respectively. Right pupil diameter was highly correlated with the left pupil diameter. We removed the left pupil diameter data throughout all the problems.

Classifying RTs as attentive, semi- attentive, or inattentive: We classify RT responses into three classes: attentive, semi-attentive, and inattentive using the three labeling methods listed in Table 1. There were no overlapping RTs under the KNN method. However, in the GMM method, the original inattentive cluster had 35 RTs that overlapped with the attentive cluster. Since this overlap was a very small percentage of all the RTs (0.00074%), we re-assigned these 35 RTs to the attentive cluster. The naive method sorts the RTs in ascending order and divides the RTs into three equal parts. The first, second, and third 33.33% of the RTs will be labeled attentive/semi-attentive and inattentive respectively. Under this method, the data will be (almost) balanced, meaning each label (by construction) will have the same number of RTs. The RTs that overlapped between labels were assigned to the label with the highest frequency.

Feature selection method for EC-classification problems: Least Absolute Shrinkage and Selection Operator (LASSO) regression and Ridge regression are traditional regularization techniques used to shrink the coefficients of a model with the goal of avoiding overfitting. LASSO regression uses a *l1* norm penalty in its cost function whereas Ridge regression uses a *l2* penalty its cost function.

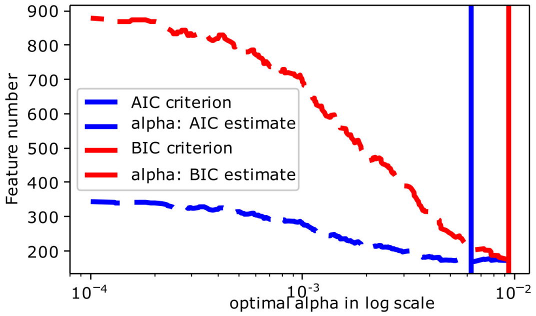

Figure 2: **Illustrating AIC and BIC alphas for the binary goal problem**. Vertical lines indicate optimal alpha values for the Akaike Information Criterion (blue) and for the Bayesian Information Criterion (red).

LASSO regression cost function:

$$\frac{1}{(2 \times \text{number of samples})} * \|y - Xw\|_2^2 + \alpha * \|w\|_1 \tag{1}$$

Ridge regression cost function:

$$\|y - Xw\|_2^2 + \alpha * \|w\|_2^2, \tag{2}$$

where α is a non-negative regularization parameter, w are the regression coefficients, y represents the observed data, and Xw the predicted values using the w coefficients.

AIC and BIC for model comparison: The Akaike Information Criterion (AIC) and the Bayesian Information Criterion (BIC) are methods for comparing models. The lower the AIC/BIC, the better the model is. A good model will use few features to yield high accuracy. In sklearn we used the LASSO with AIC and BIC to obtain the optimal alphas for each problem (see Fig S2) and the coefficients of features for each problem. Then we normalized the absolute values of the features coefficients (obtained by using the optimal AIC and BIC alpha values) separately so that we can compare them. Then we summed them up and ranked them according to their combined sum to provide a score for each feature. We ranked the features using this score to obtain a sorting of the features. This method did not provide us with enough features (11,1,4,1,3 and 5 for $C_{EC}^{group}[1, \ldots, 6]$ respectively). Therefore we used ridge regression to acquire more features by using the optimal α obtained through cross validations. We ran ridge regression with this optimal α and sorted the features according to their coefficient values. Then we appended these sorted features (from high importance to low importance) to the end of the LASSO features to expand the number of features. For some problems, there was an overlap of features obtained by both the methods, and in that case, we dropped the repeated ridge features when appending. The number of ridge regression features appended was capped at 2 to keep a low number of features.

Balanced accuracy and AIC values for different features: Step six in Figure 2 uses the lazy predict wrapper to select the best model (number of features and the classifier) for the problems $C_{EC}^{group}[1, \ldots, 6]$. Tables S1-6 list how the balanced accuracy and AIC values change according to the number of features selected for each problem. The balanced accuracy and the AIC values reported

here need to be interpreted as upper bounds since only 5 manual cross-validations have been used to attain these values.

Balanced accuracies and AIC values for different resolutions (experimental conditions classification problems): We investigated how coarser measurements can classify the subjects in problems $C_{EC}^{group}[1,\ldots,6]$. Table S7 reports the number of features selected, the balanced accuracy, standard deviation, the best classifier, and the AIC values for each resolution and each classifier problem $C_{EC}^{group}[1,\ldots,6]$.

Table 1: $C_{EC}^{group}[1]$: Binary goal

No. of features	Classifier	Balanced Accuracy	AIC
1	Logistic Regression	0.65	-331.26
2	SGD Classifier	0.69	-346.48
3	SVC	0.70	-352.56
4	Ridge Classifier CV	0.75	-374.91
5	Linear Dis. Analysis	0.75	-375.16
6	Ridge Classifier CV	0.76	-374.85
7	SVC	0.75	-366.86
8	SGD Classifier	0.78	-387.46
9	Linear Dis. Analysis	0.76	-366.24
10	Ridge Classifier CV	0.77	-369.80
11	Ridge Classifier CV	0.78	-375.47

Table 2: $C_{EC}^{group}[2]$: Binary feedback

No. of features	Classifier	Balanced Accuracy	AIC
1	Gaussian NB	0.77	-229.23
2	Ridge Classifier	0.78	-231.64
3	Linear DIs. Analysis	0.79	-237.66

Table 3: $C_{EC}^{group}[3]$: Binary reward

No. of features	Classifier	Balanced Accuracy	AIC
1	Label Propagation	0.65	-273.72
2	Quadratic Dis. Analysis	0.70	-293.08
3	Nearest Centroid	0.66	-276.34
4	Linear SVC	0.71	-293.24
5	Quadratic Dis. Analysis	0.74	-302.88

Table 4: $C_{EC}^{group}[4]$: Tertiary goal

No. of features	Classifier	Balanced Accuracy	AIC
1	Ridge Classifier	0.51	-284.55
2	Calibrated Classifier CV	0.52	-284.07

Table 5: $C_{EC}^{group}[5]$: Tertiary reward

No. of features	Classifier	Balanced Accuracy	AIC
1	K-Neighbors Classifier	0.52	-239.99
2	Ridge Classifier CV	0.57	-249.89
3	Ridge Classifier CV	0.56	-244.60
4	Logistic Regression	0.57	-246.82
5	Linear SVC	0.61	-254.26

Table 6: $C_{EC}^{group}[6]$: All subgroups

No. of features	Classifier	Balanced Accuracy	AIC
1	Ada Bosst Classifier	0.25	-463.87
2	Gaussian NB	0.25	-460.54
3	SVC	0.27	-467.56
4	SVV	0.27	-468.05
5	Ridge Classifier CV	0.28	-469.74
6	Linear SVC	0.28	-467.11

Table 7: **Balanced accuracies and resolution.** This table depicts how the balanced accuracies reduce as the resolution increases and the measurements become coarser for problems $C_{EC}^{group}[1,\ldots,6]$.

Problem		No. of Features	Classifier Method	Balanced Accuracy	Standard Deviation	AIC
6[2]***Res. 10**	[1]	5	Linear dis. analysis	**0.6**	0.16	-629.75
	[2]	2	Passive agg. Classifier	**0.68**	0.22	-396.38
	[3]	3	Ridge clas. CV	**0.52**	0.17	-483.54
	[4]	2	Extra tress classifier	**0.38**	0.12	-582.07
	[5]	2	Calibrated class. CV	**0.38**	0.11	-478.52
	[6]	8	Ridge classifier	**0.23**	0.1	-1067.21
6[2]* **Res. 20**	[1]	6	Linear dis. analysis	**0.62**	0.15	-640.57
	[2]	3	Linear SVC	**0.53**	0.1	-385.13
	[3]	2	Ada boost classifier	**0.63**	0.18	-526.77
	[4]	2	Linear dis. analysis	**0.36**	0.13	-566.74
	[5]	1	Label spreading	**0.33**	0	-487.29
	[6]	5	Linear SVC	**0.17**	0.06	-1054.32
6[2]* **Res. 35**	[1]	1	Label propagation	**0.5**	0	-628.81
	[2]	3	Ridge classifier CV	**0.54**	0.15	-384.83
	[3]	3	Ada boost classifier	**0.55**	0.17	-496.47
	[4]	2	Linear dis. Analysis	**0.38**	0.13	-574.12
	[5]	1	Linear SVC	**0.33**	0	-487.29
	[6]	3	Calibrated class. CV	**0.18**	0.06	-1064.04
